# Closed circuit xenon delivery for 72h in neonatal piglets following hypoxic insult using an ambient pressure automated control system: Development, technical evaluation and pulmonary effects

**DOI:** 10.1371/journal.pone.0224447

**Published:** 2020-01-21

**Authors:** John Dingley, Satomi Okano, Richard Lee-Kelland, Emma Scull-Brown, Marianne Thoresen, Ela Chakkarapani

**Affiliations:** 1 Department of Anaesthetics ABM University Health Board, Swansea and College of Medicine, Swansea University, Swansea, Wales, United Kingdom; 2 Translational Health Sciences, Bristol Medical School, University of Bristol, Bristol, England, United Kingdom; 3 Translational Health Sciences, Bristol Medical School, University of Bristol, Bristol, England, United Kingdom; 4 Department of Physiology, Institute of Basic Medical Sciences, University of Oslo, Oslo, Norway; University of PECS Medical School, HUNGARY

## Abstract

**Background:**

Therapeutic hypothermia (TH) for 72h is the standard treatment following neonatal encephalopathy (NE). However, one-third do not benefit and adjunctive therapies are urgently needed. Xenon enhances neuroprotection with TH when administered at 50% concentration within 5hours of hypoxia in experimental studies. Delayed initiation (~10 hours of age) of 30% xenon for 24 hours during TH did not improve early adverse biomarkers in a clinical trial of Xenon+TH vs TH. After hypoxia-ischemia, excitotoxic injury via N-methyl-D-aspartate receptor overactivation lasts days. Since xenon partially inhibits this receptor, we hypothesised that giving 50% xenon throughout the entire 72h TH and rewarming periods would enhance neuroprotection. Xenon costs $30/litre, so a closed-circuit breathing system is desirable with automated fresh gas delivery.

**Methods:**

Seven mechanically ventilated newborn pigs were randomized to receive 50% inhaled xenon for 72h during hypothermia (rectal-temperature 35°C) and subsequent rewarming following a global hypoxic-ischemic insult (XeHT, N = 4) or under normothermia for 72h (rectal-temperature 38.5°C) following sham insult (XeNT, N = 3). An automated fresh gas delivery system injected oxygen/air/xenon boluses into a closed-circuit based on measured gas concentrations.

**Results and discussion:**

Median (IQR) xenon consumption was 0.31 L/h (0.18, 0.50) and 0.34L/h (0.32, 0.49) for hypothermic and normothermic groups respectively, 0.34L/h (0.25, 0.53) overall. 92% of 9626 xenon and 69% of 9635 oxygen measurements were within 20% variation from targets. For xenon concentration, the median absolute performance errors for the XeHT and XeNT groups were 6.14% and 3.84% respectively and 4.31% overall. For oxygen these values were 13.42%, 15.05% and 12.4% respectively. There were no adverse pulmonary pathophysiology findings. Clinical problems over the total period included three related to sensors, seven breathing system leaks, ten partial and one complete tracheal tube occlusion episodes.

**Conclusion:**

The automated controller functioned as intended maintaining an inhaled xenon concentration close to the 50% target for 72-78h at a xenon cost of $11.1/h.

## Introduction

Xenon, an anesthetic noble gas, is also a neuroprotectant reducing hypoxic brain injury following birth asphyxia (hypoxic-ischemic encephalopathy, HIE) and stroke in newborn pre-clinical models [1–5]. There is currently a large international clinical trial in progress investigating neuroprotective effects of xenon in combination with cooling after cardiac arrest, based on a favourable smaller trial [6–8]. It is also showing promise in pre-clinical in-vivo neuroprotection studies of other brain injury models such as sub-arachnoid haemorrhage and traumatic brain injury [9–12]. Xenon may protect other organs such as the heart [13–15] and kidney [16–18], and at present it is licensed for anesthesia in some European countries while these other possible uses are the subject of ongoing medical research. The standard treatment for HIE is therapeutic hypothermia (TH) i.e. cooling for 72hours (h), in order to reduce death or disability [19]. Currently, despite TH, 11% of cooled infants still die and a further 22% of survivors develop disability [20]. To improve the neurodevelopmental outcomes, xenon is being investigated as a potential adjunct neuroprotective agent in clinical trials [21] Pre-clinical data has indicated that xenon commenced within 5h of the hypoxic-ischemic insult at 50% concentration enhanced hypothermic neuroprotection [2,3,22]. In the TOBY-Xe clinical trial, a combination of xenon and whole body cooling did not alter early MRI biomarkers of adverse neurodevelopment versus cooling alone. However, the concentration of xenon given at 33% was lower than, and the time delay between birth and xenon delivery of approximately 10 hours was much greater than those used in most pre-clinical studies, so this finding is not entirely unexpected [23]. In general, regardless of the neuroprotection modality being investigated, it is highly desirable to have the shortest possible delay before treatment onset.

Following hypoxia-ischemia, glutamate accumulates in the synaptic cleft causing excessive activation of the N-methyl-D-Aspartate (NMDA) receptor leading to excitotoxicity. The excitotoxic cascade of brain injury continues for days leading to long term disabilities [24]. As xenon is an inhibitor of NMDA receptors at the glycine-co-agonist site, administering xenon for the entire 72h duration of TH may reduce the excitotoxicity and enhance the neuroprotection [25,26]. In addition there are other molecular effects of xenon that may be involved in its neuroprotective effect such as activation of two-pore-domain potassium channels TREK-1, inhibition of calcium adenosine triphosphate pump activity, antiapoptotic and stimulation of norepinephrinergic neurons [27–29]. Xenon costs approximately $30/L, and so use of a circle breathing system even with low fresh gas flows can still be very expensive over a long administration period. However as tissue uptake of xenon via the lungs is extremely slow, a closed-circuit breathing system would be an ideal means to maximally exploit this property to minimise fresh xenon requirements [29–33].

Delivering xenon for long periods via a closed-circuit breathing system under manual control has proved to be possible, however an automated breathing gas control system would be more desirable [34]. It may also be desirable to supply this control system with fresh xenon at ambient pressure, in order to largely eliminate the very high costs incurred when what would normally be considered trivial leaks are present in systems supplied for long periods with pressurised gas. The neonatal pig model of global hypoxic-ischemic encephalopathy (HIE) simulates the pathophysiology, brain and organ injury including lung dysfunction and injury of the human condition [35,36] and has been used during the preclinical development of hypothermia as a neuroprotective intervention following NE.

Here, we developed an automated gas administration and control device with an ambient pressure gas supply, for use with a single use closed circuit xenon breathing system. We evaluated its ability to consistently maintain xenon at user-set target concentrations and investigated the pulmonary effects including gas exchange and lung pathology of administering xenon for 78h with 72h concurrent hypothermia and 72h without hypothermia in the newborn pig global HIE model.

## Materials and methods

These experiments were carried out under Home Office License (project license no: 3003332) in accordance with UK guidelines and approved by the Animal Welfare Ethical Review Board at the University of Bristol. This manuscript adheres to the applicable Equator guidelines.

**Fig 1:**
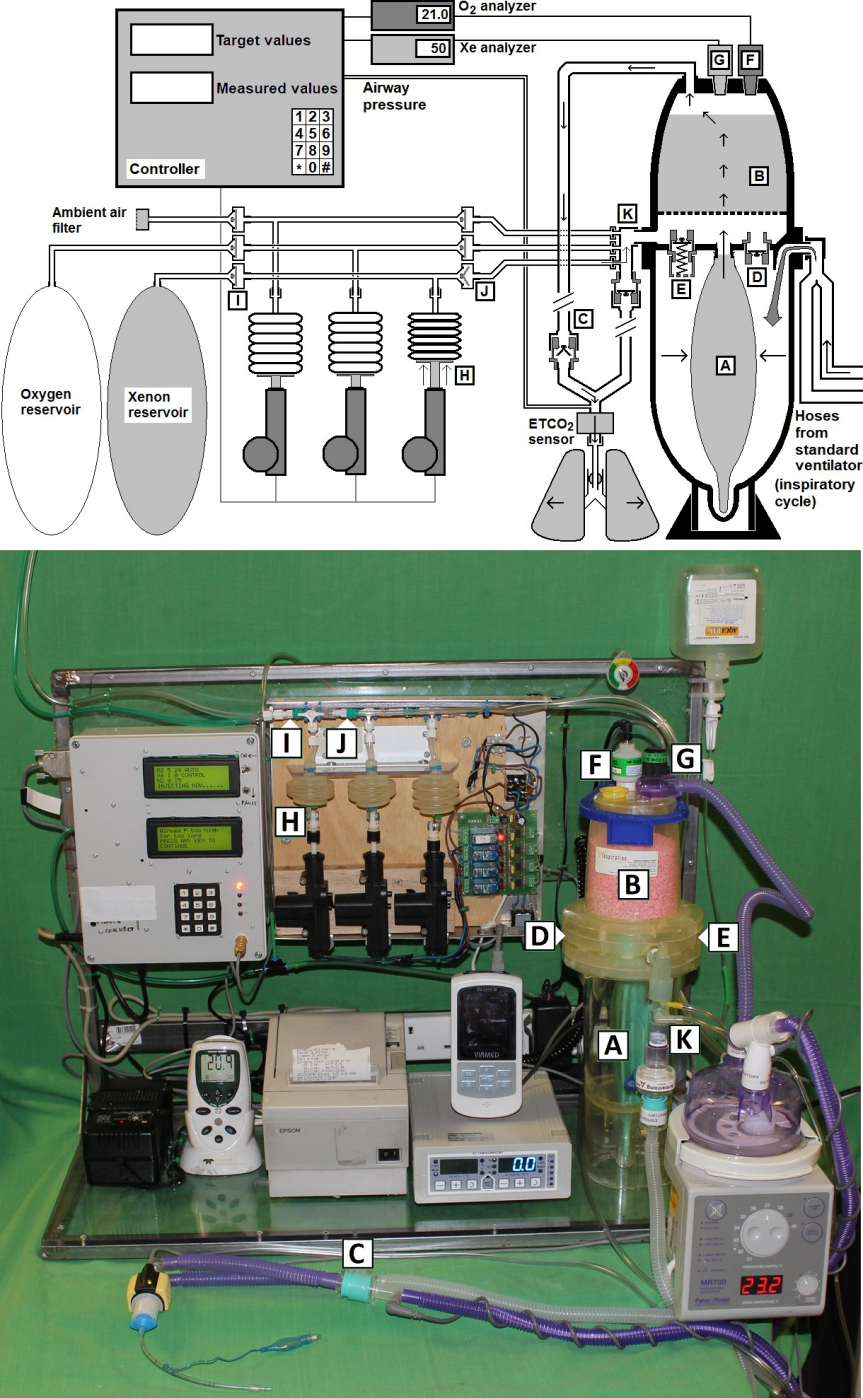
Diagram and corresponding image of apparatus. Upper: Combination of closed circle (left side) and automated gas controller (right side). Oxygen driving gas from a conventional ventilator enters the lower chamber during inspiration, compresses bag (A) causing gas to flow through soda-lime (B) in the upper chamber, down an inspiratory hose to the lungs via a check valve (C). An oxygen substitution valve (D) permits oxygen to bypass into the upper chamber in the event of any large leak or failure of the automated gas controller. There is also an overpressure spill valve (E) for safety. The automated controller reads data from oxygen (F) and xenon (G) sensors and, during expiration as detected via airway pressure changes, injects fresh gas boluses as required. In this example, linear actuator (H) has drawn 18ml xenon from the xenon reservoir via unidirectional valve (I) into its small bellows and is injecting it via unidirectional valve (J) into the expiratory limb (K). The inspiratory hose also contains a humidifier and heated wire (not shown). Lower: Photograph of automated delivery system with labels corresponding to those of upper diagram. Monitors and additional devices present are, from left to right; oxygen monitor, backup data printer, xenon analyser with end-tidal carbon dioxide monitor mounted above it and a humidifier with heated wire in the inspiratory hose.

To exploit the known low uptake of xenon via the lungs and the small size of neonates compared to adults, a closed-circuit breathing system was used to minimise xenon consumption [31]. This single-use circuit has been previously used by us in experimental and human neonatal clinical trials with manual addition of aliquots of fresh gas [34]. For this investigation an automated gas controller was developed to monitor the recirculating breathing gas composition of this circuit and inject oxygen, air or xenon as required to bring the circulating gas mixture towards user-set target values. Its main component parts were; sensors for measurement of gas concentrations and airway pressure, actuators to deliver individual gases to the circle, custom written control software running on a microcontroller (a small embedded computer), and a user interface “Fig 1”.

### Sensors and data collection

A conventional fuel cell oxygen analyser (AX300, Viamed Ltd., Keighley, UK) output the measured oxygen fraction as a voltage with a range of 0 – 1V (0–100%) to the microcontroller which converted this information into a digital form for further processing. Xenon has a thermal conductivity markedly lower than that of other gases in the breathing system. Xenon concentration was measured by a thermal conductivity analyser (GKM-03-INSOVT, St. Petersburg, Russia. Obtained via Alfa-Impex Oy, Finland) with an RS232 serial data output to the microcontroller. A pilot tube from the tracheal tube connector was connected to a pressure transducer (MPXV7002DP, NXP Semiconductor, Austin Texas, US) which was continually monitored by the microcontroller, allowing detection of and differentiation between, inspiratory and expiratory cycles as well as circuit disconnection.

### Actuators

Three linear actuators (Universal heavy duty actuator, Candy Electronics Technology Co., Guangdong, China), were arranged so that under control of the microcontroller, they would compress and then release, each of a set of three miniature silicone bellows (Pediatric test lung bellows, Drägerwerk, Lübeck, Germany) fitted with inlet and outlet unidirectional valves (Infuvalve®, B Braun, Melsungen, Germany). Each therefore acted as a miniature gas pump for xenon, oxygen or air, adding 18ml of gas to the breathing circuit each time the respective actuator operated. An ambient pressure reservoir bag of oxygen and xenon was attached to two of the bellows inlets while the third inlet was open to air via a filter, allowing it to draw in, and inject, ambient air when required. This means of fresh gas supply had previously been found to work with a manually controlled xenon delivery system in neonates [37]. The outlets of these gas pumps entered the expiratory limb of the circle system, allowing fresh gases the maximum opportunity to mix with breathing system gases before entering the lungs.

### Microcontroller and control algorithm

**Fig 2:**
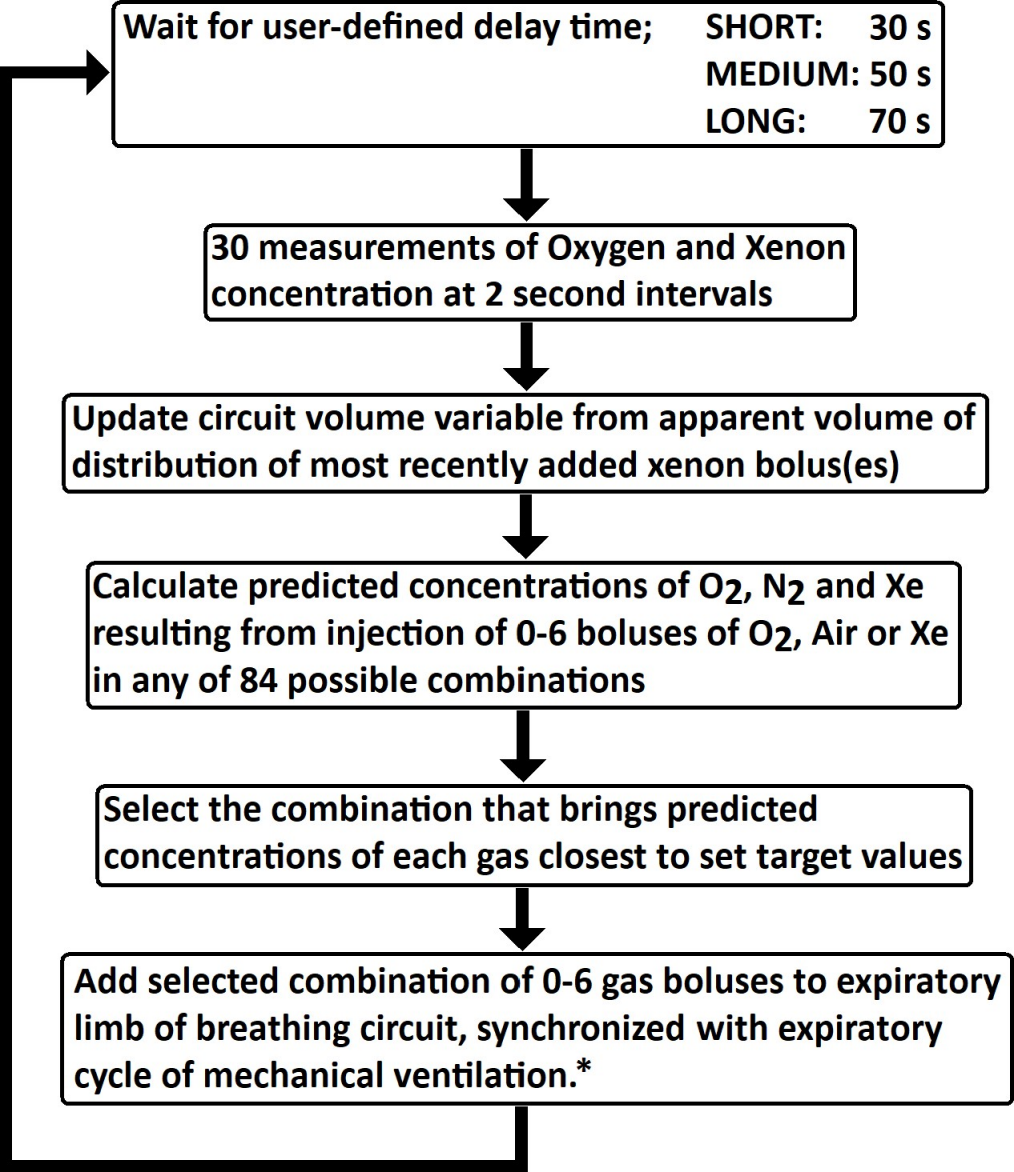
Algorithm used by the automated controller for injection of boluses of fresh oxygen, air or xenon at ambient pressure into the closed-circuit breathing system. * The microcontroller continually measured the airway pressure in the background in order to firstly present an alarm if it fails to detect normal cycling of airway pressure and secondly to allow injection of each bolus of fresh gas to be performed in synchrony with the expiratory cycle of mechanical ventilation.

The control algorithm is described “Fig 2”. After each fresh gas injection cycle, the controller paused for a user-selectable time interval of 30, 50 or 70s to allow mixing of gases within the circle, after which the means of 30 measurements of oxygen and xenon concentrations were taken at 2s intervals to offset any residual unevenness of gas mixing around the circle. These values were then compared with user-set target values for xenon and oxygen. Each gas injector bellows would inject an 18ml bolus of gas when activated. In a manner similar to a chess computer, the algorithm was programmed to predict the effect on the resulting combined gas concentrations of injecting all possible combinations of gas boluses (oxygen, xenon or air) up to a total of 6 boluses per cycle, in any of 84 possible combinations. These calculations required an estimate of overall system volume, a value reflecting not only the volume of the circle system and lungs but also the effect of any other losses such as patient uptake (minimal) and leaks. It would then select the combination that brought the predicted values closest to the user-set target values. The microcontroller continually monitored airway pressure which allowed it to act as a disconnect alarm but also permitted it to only inject the selected combination of fresh gas boluses during the expiratory cycle of ventilation. In any given cycle of measurement/calculation/gas injection, it would inject up to 6 boluses of fresh gas, in synchrony with expiratory cycles of the mechanical ventilator. This was done to prevent injected gas boluses adding to the set inspiratory tidal volume if a volume-controlled ventilation mode was in use. After waiting for the set mixing time, the controller would measure another set of gas concentrations, compare the previous prediction of gas concentrations against these new measured values and then adjust the overall system volume value upwards or downwards by a fraction limited to ±10% per cycle, accordingly. In this way its performance could gradually self-tune over time and also compensate for situations such as very small leaks.

### User interface

The controller unit had two 4-line back-lit liquid crystal displays. Although a long-established technology, these are easy to read in any lighting conditions. During initial set-up a series of menus was presented to the user allowing selection of the type of oxygen and xenon analysers in use, the desired mixing time delay between cycles and target xenon and oxygen concentrations, using a keypad. A toggle switch allowed the user to engage the automated controller or pause it, for example while performing tracheal tube suction. While paused, any of the above settings could be adjusted. When running, the controller displayed measured, predicted and target concentrations for all three gases, while also showing a countdown timer until the next set of measurements, the estimated system volume variable and the pattern of gas boluses selected by the algorithm in the previous cycle, e.g. 3 xenon, 2 oxygen, 1 air. Alarms were provided for circuit disconnection, high or low airway pressure, loss of signal from either gas analyzer and a low F_i_O_2_ from any cause. The estimated overall system volume variable, dependent upon the concentration response to previous injections of xenon, functioned as small leak indicator as in this event its value would increase above that typically expected.

### Animal preparation

Crossbred *Landrace/Large White* newborn term pigs were housed in a pen with their companions before and in an intensive care facility during the experiment until perfusion/fixation. All facilities were compliant with UK Home Office regulations as described previously [2]. In brief, pigs after anaesthetic induction and intubation with a 3.0mm cuffed tracheal tube, were ventilated using a neonatal ventilator (SLE 2000; SLE, Croydon, Surrey) with 1–2% isoflurane, 70% N_2_O and 28–29% O_2_ (humidified) before conversion to the xenon containing gas mixture. 10% dextrose was administered at 60mls/kg/day. Oxygen saturation was measured and also end-tidal CO_2_ via a mainstream capnograph to prevent xenon removal via any gas sampling line (VM-2500-M Mainstream Capnograph, Viamed Ltd., Keighley, UK). Oxygen saturation and end-tidal CO_2_ was maintained between 92–95% and 4.5–5.5kPa respectively. A commercial temperature control system pumped water at a variable temperature through a wrap enveloping the pigs to maintain their core temperature at a user set value (Criticool, MTRE, Yavne).

### Experiment protocol

After animal preparation (n = 7), four anesthetized and ventilated newborn pigs were randomised to receive 50% inhaled Xe via an automated delivery system for 78h during hypothermia and rewarming (rectal temperature, T_rectal_ 35°C for 72h followed by rewarming) following a global hypoxic-ischemic insult (XeHT, N = 4) and three were randomised to receive xenon during normothermia (for pigs) for 72h (T_rectal_ 38.5°C) following a sham insult (XeNT, N = 3), with background propofol and fentanyl infusions. The hypoxic-ischemic insult was applied according to an established described technique; after checking the tracheal tube cuff for an absence of leaks, the inspired oxygen fraction was reduced to between 3 and 7% to depress the background aEEG amplitude to below 7 μV for 45 minutes [30].

The automated gas control system and closed circuit was used to maintain target xenon and oxygen concentrations for this entire period. Manual partial flushes of the breathing system with fresh gas were carried out at intervals of 8h.

After the end of intervention, pigs were extubated once they were able to breathe spontaneously. After a further 12h normothermic survival, pigs underwent perfusion fixation of brain under deep anaesthesia. We conducted autopsy and removed the lungs, which were fixed in 4% formaldehyde. Two sections from the three lobes of the right lung and two lobes of the left lung were stained with haematoxylin and eosin and, and the elastic fibres were stained with Verhoeff’s Van Gieson stain [38]. Two slides from each lobe of the lung (right lung: 3 lobes and left lung: 2 lobes) were scored qualitatively for the presence of histological abnormalities, intra-alveolar fibrin, hyaline membranes, inflammation and oedema. The pathologist who was blinded to the intervention received by the piglets categorically scored the presence of these abnormalities as yes (1) or no (0).

### Statistical analysis

We utilised the pooled-data approach to assess the performance of the system [39,40].

#### Percentage performance error

Percentage performance error (PPE) was defined as the percentage difference between each measured gas concentration in the breathing system and the target value for that gas. The PPE for the ith piglet at the jth measured value versus the set target value at that time was calculated as follows:

$${\mathrm{PPE}}_{\mathrm{ij}}=\frac{\left({\mathrm{measured}\;\mathrm{gas}\;\mathrm{concentration}}_{\mathrm{ij}}-{\mathrm{target}\;\mathrm{gas}\;\mathrm{concentration}}_{j}\right)}{{\mathrm{target}\;\mathrm{gas}\;\mathrm{concentration}}_{i}}\;X\;100$$

#### Median absolute performance error

Median absolute performance error (MDAPE) is a measure of inaccuracy and indicates the absolute magnitudes of the differences between measured and target gas concentrations.

MDAPE_i_ = median{|PPE_ij_|, j = 1,……,N_i_}

$$\mathrm{MDAPE}=\frac{1}{\sum_{i=1}^{M}N_{i}}\times \sum_{i=1}^{M}\left(N_{i}^{x}\;MDAPEi\right)$$

with Ni being the number of values of |PPE| for the ith piglet and M being the number of piglets in the study.

#### Median performance error

Median performance error (MDPE) is a measure of bias and indicates whether the differences between measured gas concentrations were systematically above or below the target gas concentrations.

MDPE_i_ = median{PPE_ij_j = 1,…….,N_i_}

$$\mathrm{MDPE}=\frac{1}{\sum_{i=1}^{M}N_{i}}\times \sum_{i=1}^{M}(N_{i}^{x}\;{MDPE}_{i})$$

#### Wobble

Wobble measures how much PPE fluctuates around the MDPE with time, for each piglet (i.e. intrasubject PPE variability).

WOBBLE_i_ = median {|PPE_ij_−MDPE_i_|,j = 1,…….,N_i_}

$$\mathrm{WOBBLE}=\frac{1}{\sum_{i=1}^{M}N_{i}}\times \sum_{i=1}^{M}(N_{i}^{x}\;{WOBBLE}_{i})$$

All calculations were performed using Microsoft Office Excel, 2016, version 1803 (Microsoft Corporation, Redmond, WA, USA).

## Results

**Fig 3:**
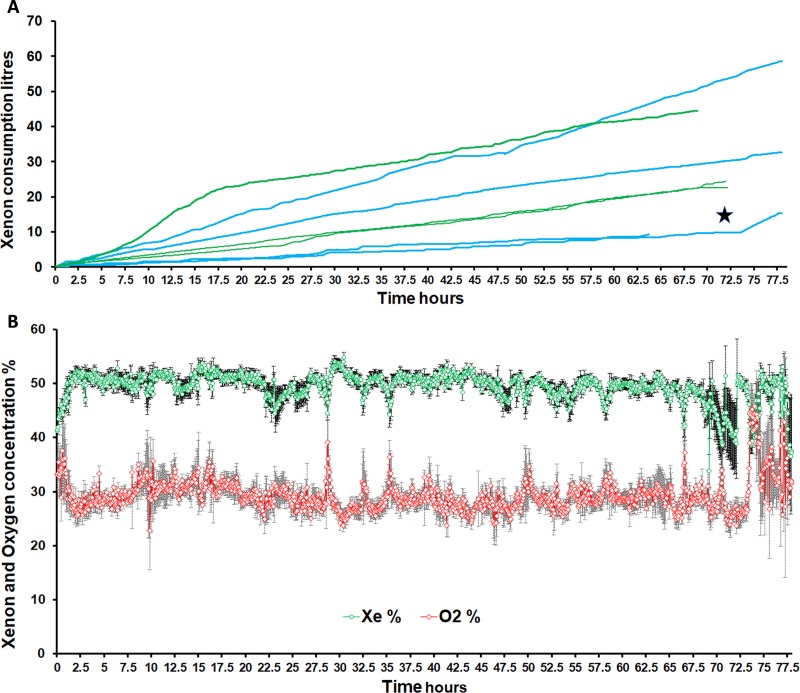
Mean (SEM) xenon and oxygen concentrations with cumulative xenon consumption shown above on same time scale. On xenon consumption chart: Green lines represent Normothermia animals while Blue lines represent Hypothermia (72h) followed by rewarming. In one case*, the xenon consumption rate can be seen to suddenly increase due to the presence of a leak, which was compensated for by the gas controller. One xenon-hypothermia experiment was stopped early, as the animal underwent terminal perfusion fixation due to refractory hypotension. In the xenon oxygen concentration graph, green circles denote xenon concentration and red circles denote oxygen concentration.

The piglets (n = 7) had a median (IQR) weight of 1.53 kg (1.41, 2.09), with a median (IQR) age of 14.0 hours (9.3, 19.5) from birth. 57% were males. During the automated control of breathing, we acquired a total of 9626 xenon and 9635 oxygen artefact free concentration measurements over a median (IQR) total running period of 68.6 hours (67.1, 70.7). The median (IQR) hourly Xe consumption in the XeHT group was 0.31 L/h (0.18, 0.50) and in the XeNT group was 0.34L/h (0.32, 0.49) (Fig 3) and the overall consumption was 0.34L/h (0.25, 0.53). The maximum and minimum total xenon consumption over 72 hours for the seven pigs were 53.5 L and 9.3 L. The slopes of xenon consumption in the XeHT pigs were 7.435, 1.358, 6.334 and 2.382, whilst the slopes in the XeNT group were 1.539, 2.977 and 3.135. The concentrations of xenon and oxygen in the breathing gas mixtures are also shown in (Fig 3). 92% of 9626 xenon measurements and 69% of 9635 oxygen measurements were within 20% variation from target values respectively. Among the 7 pigs, 5 reached the target xenon concentration of 50% within 50 mins, 1 pig reached target by 60 min and the other pig reached target by 100mins. Mean (SD) xenon concentration during the 72hour cooling period was 49.6% (4.52) and the mean(SD) oxygen concentration during the 72-hour cooling period was 28.9% (5.06). (Fig 3) During rewarming, the mean (SD) xenon concentration was 48% (3.53) and the mean (SD) oxygen concentration was 33.7% (8.1). In the final hour of rewarming, xenon concentration was reduced to a mean (SD) of 37.3% (26), whilst maintaining a mean(SD) oxygen concentration of 31.8% (9.75). (Fig 3)

**Fig 4:**
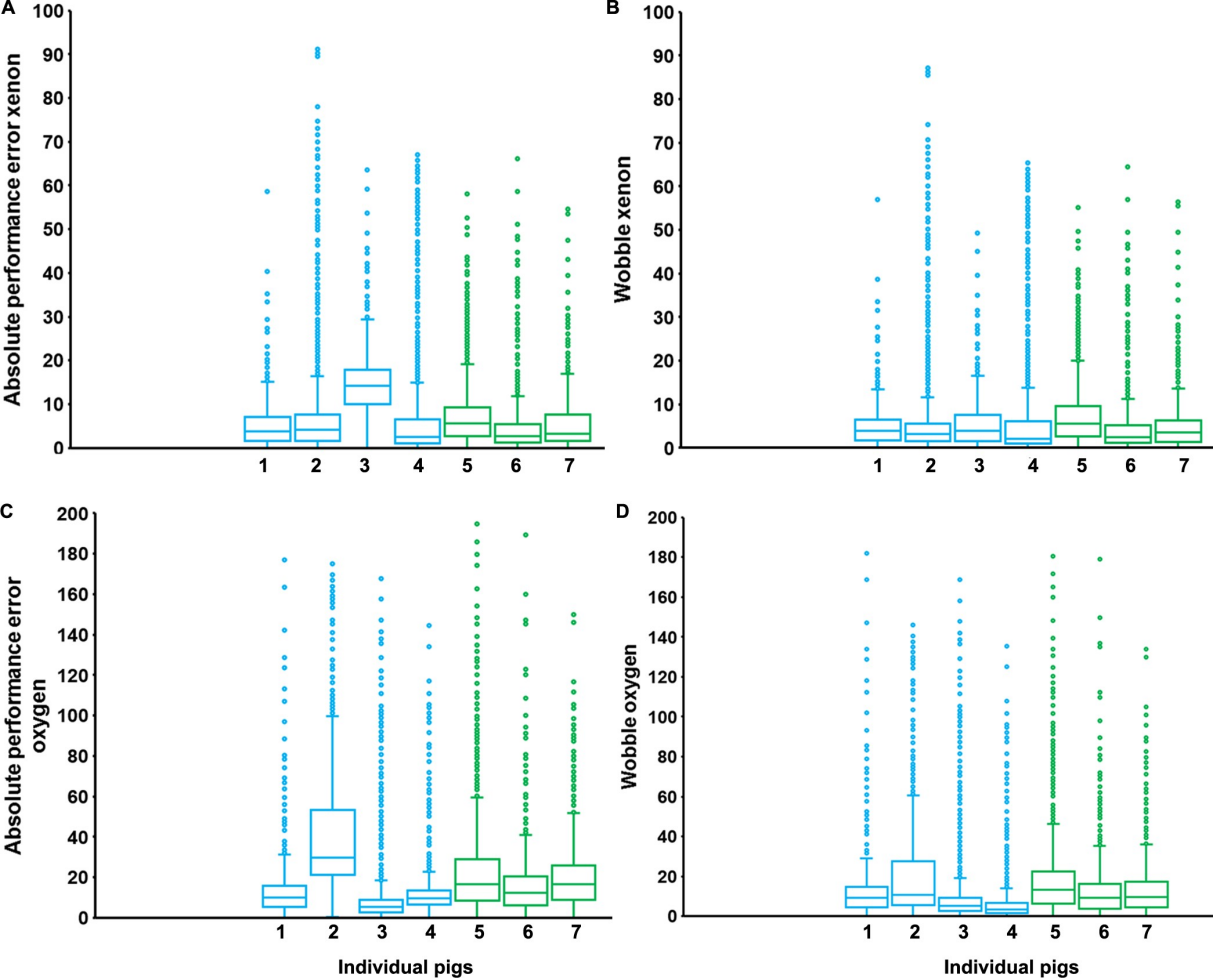
Xenon and oxygen control parameters. A) Boxplot Absolute PPE Xenon. Represents absolute magnitude of the differences between measured and target gas concentrations. B) BoxplotWobble for Xenon. Represents the fluctuation in percentage performance error around the median performance error (MDPE) for each subject. C) Boxplot Absolute PPE Oxygen. Represents absolute magnitude of the differences between measured and target gas concentrations. D) Boxplot Wobble for Oxygen. Represents the fluctuation in percentage performance error around the median performance error (MDPE) for each subject. Box plot represents the minimum, interquartile range, median and the maximum values. The outliers are shown as blue filled circles for the XeHT group and green filled circles for the XeNT group.

With regard to the performance of the system, the absolute performance error and wobble are described in (Fig 4). Median (IQR) of absolute performance error for xenon concentration in the XeHT group were 3.7 (1.56, 6.98), 4.2 (1.56, 7.62), 14.1 (9.91, 17.75) and 2.4 (0.96, 6.55) and in the XeNT group were 5.5 (2.69, 9.23), 2.7 (1.15, 5.47) and 3.3 (1.48, 7.69). The distribution of outliers in the absolute performance error for xenon concentration were similar between the XeHT and the XeNT group with maximum values of 58.6, 91.1, 63.4 and 67.7 in the XeHT group and 58.1, 66.2 and 54.5 in the XeNT group. (Fig 4) Median (IQR) of absolute performance error for oxygen concentration in the XeHT group were 10 (5.33, 15.83), 29.6 (21.36, 53.2), 5.3 (2.5, 8.92) and 9.58 (6.66, 13.33) and in the XeNT group were 16.5 (8.4, 29.13), 12.2 (6.15, 20.38) and 16.4 (8.8, 26). The distribution of outliers of absolute performance error for oxygen concentration were similar between the XeHT and the XeNT groups with maximum values of 176.8, 175.2, 167.8, 144.5 in the XeHT group and 194.8, 189.2 and 150 in the XeNT group. “Fig 4” Median (IQR) wobble for xenon concentration in the XeHT group were 3.8 (1.76, 6.47), 3.2 (1.5, 5.6), 3.8(1.55, 7.58) and 2.1 (0.96, 6.15) and in the XeNT group were 5.5 (2.69, 9.61), 2.5 (1.09, 5.19) and 3.5 (1.34, 6.34). The outliers for the wobble were similarly distributed between the XeHT and the XeNT group with maximum values of 56.8, 87.1, 49.3 and 66.0 in the XeHT group and 55.1, 64.4 and 56.4 in the XeNT group. “Fig 4” Median (IQR) wobble for oxygen concentration in the XeHT group were 9.3 (4.58,14.64), 10.8 (5.6, 27.6), 5.3 (2.49, 9.28) and 3.3 (1.57, 6.66) and in the XeNT were 13.3 (6.31, 22.49), 9.2 (3.7, 16.37) and 9.6 (4.4, 17.2). The distribution of outliers was similar between the XeHT and the XeNT group with a maximum wobble of 181.8, 146, 168.5 and 135.4 in the XeHT group and 180.3, 178.8 and 134 in the XeNT group. The pooled estimates of xenon concentrations for MDAPE, MDPE and Wobble for the XeHT group were 6.14, -5.47 and 3.21 and for the XeNT group were 3.84, -2.24 and 3.86 with values for both groups combined of 4.31, -3.26 and 3.42 respectively. The pooled estimates of oxygen concentrations for MDAPE, MDPE and Wobble for the XeHT group were 13.42, 8.36 and 6.98 and for the XeNT group were 15.05, 13.64 and 10.7 with values for both groups combined of 12.4, 9.62 and 7.86 respectively.

As expected, when ventilating animals approximately half the weight of a term human neonate, with cuffed tracheal tubes, for such an extended period, via a closed-circuit breathing system, there were a number of practical clinical problems requiring intervention. Although not necessarily directly related to the electronic control system, for example the development of a small breathing system leak, the controller would then automatically try to compensate for such anomalies so affecting its overall performance, creating variability between animals in the overall xenon consumption. These events are summarised in (Table 1).

**Table 1:** Summary of main clinical problems encountered classified by type.

Event Type	Frequency
Oxygen sensor failure.	3
Failure of sensor communication with gas controller.	2
Oscillation of F_i_O_2_ below 0.21 where this was due to the target mean F_i_O_2_ being set too low.	8
Tracheal tube partial occlusion with secretions.	10
Complete tracheal tube occlusion requiring replacement.	1
Small breathing system leak	5*
Large breathing system leak requiring complete breathing system replacement.	2
Low F_i_O_2_ due to empty oxygen reservoir bag.	1

(* In two of these instances the controller compensated for the gas leak and the situation was monitored by the operator who did not need to manually intervene. In the remainder the leak was physically corrected by the operator).

**Fig 5:**
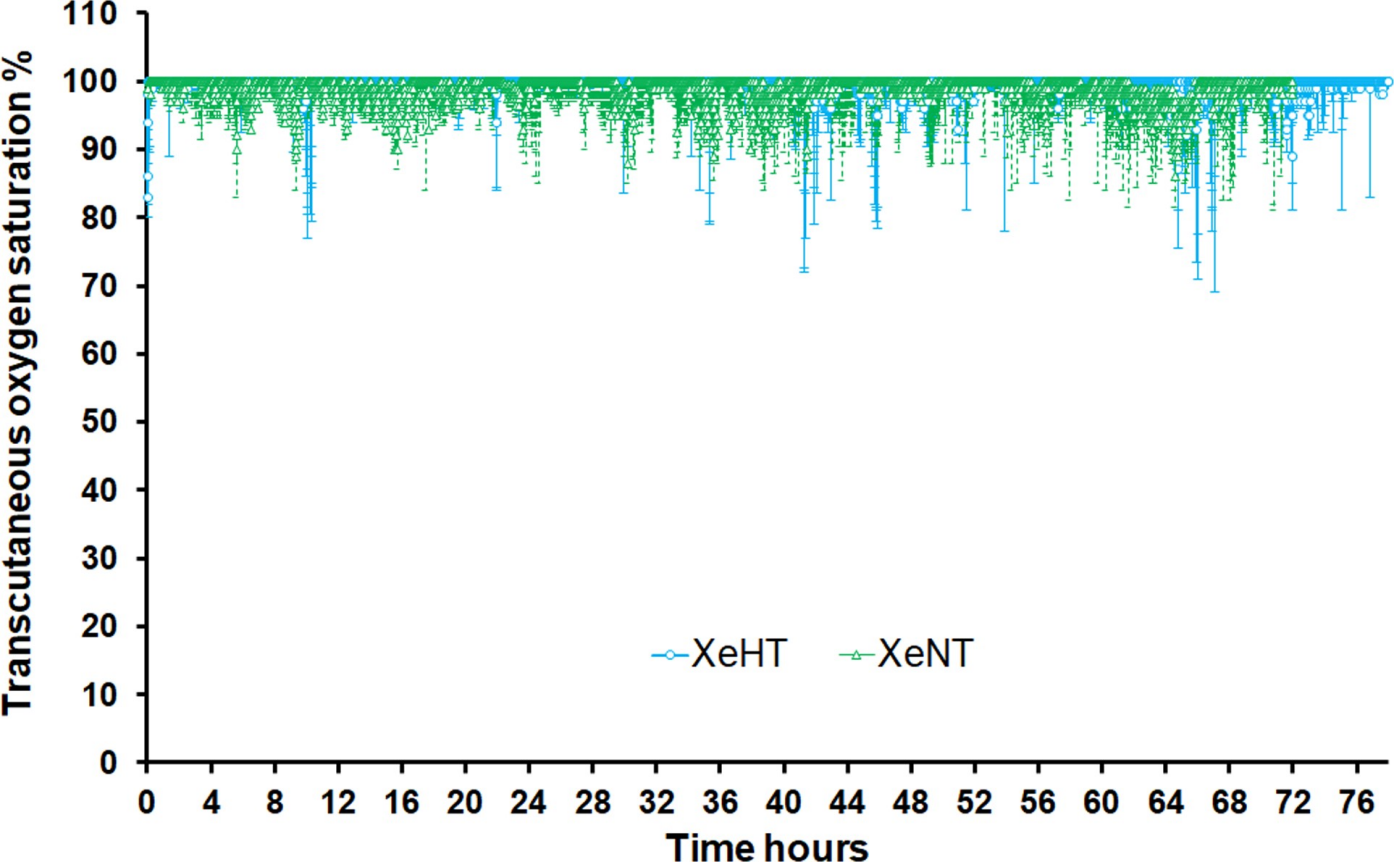
Transcutaneous oxygen saturation. Median and 25^th^ percentile data of transcutaneous oxygen saturation every minute against time. Green colour represents XeNT group and blue colour represents the XeHT group. The error bars are the 25^th^ percentile.

**Fig 6:**
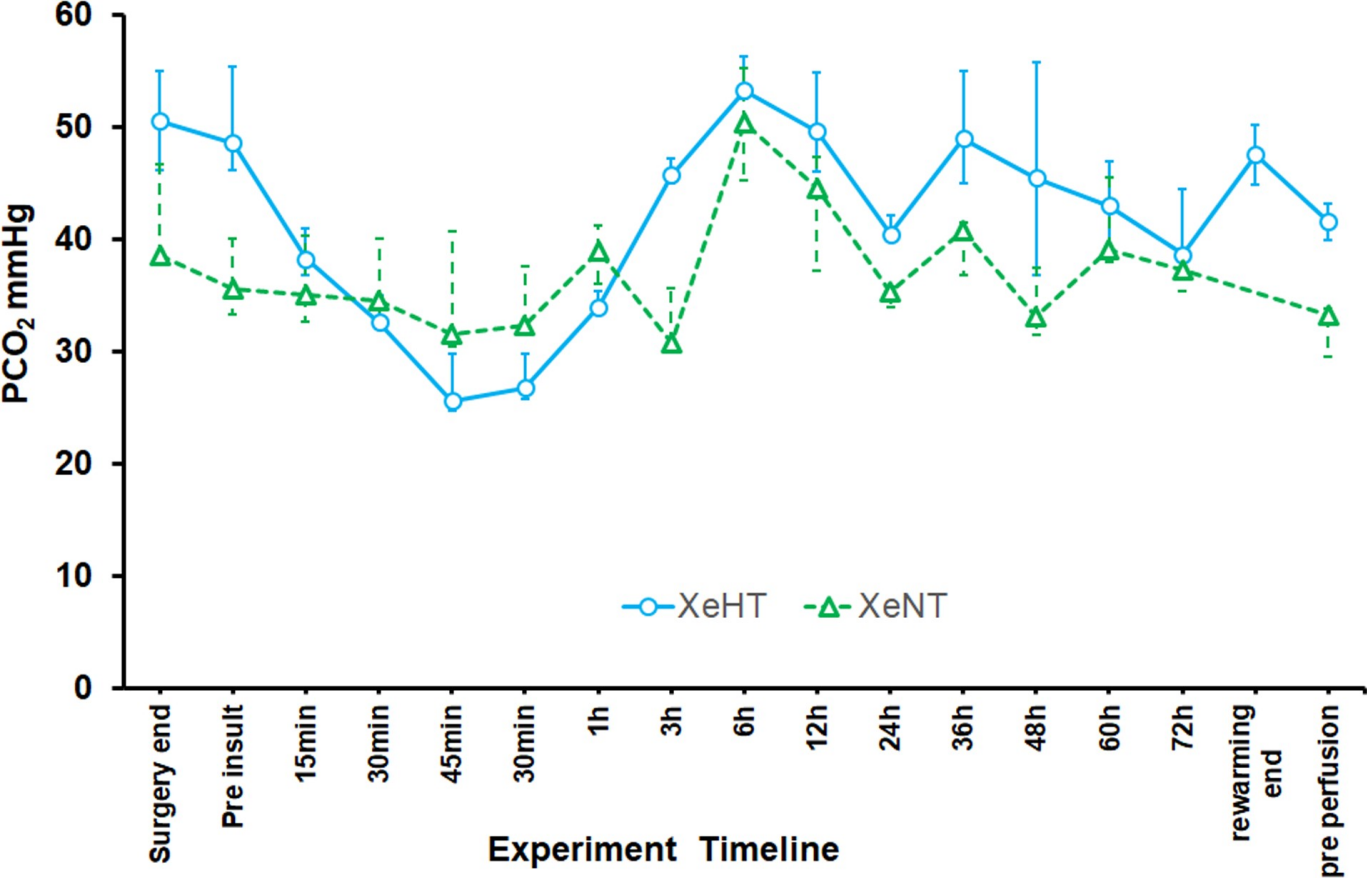
Arterial partial pressure of carbon dioxide (PCO_2_)Median (IQR) of PCO_2_ against time. Surgery end and Pre insult indicates the baseline values. The hypoxic-ischemic insult lasted for 45 mins including the time points 15min 30min and 45 min. Blue circle indicates the XeHT group and the green triangle represents XeNT group.

The transcutaneous oxygen saturation remained stable during the xenon administration period “Fig 5”. The saturation data is presented for every minute in “Fig 5”. Only 0.5% of the measurements were < 80% and 0.1% were < 70%. Arterial partial pressure of CO2 reduced from a median (IQR) of 41.7mmHg (36.4, 47.6) before hypoxic-ischemic insult to 29.3mmHg (25.7, 35.6) at the end of hypoxic ischaemic insult. The PCO2 was maintained higher during the cooling period in the XeHT group at a median (IQR) of 45.3mmHg (33.9, 53.1) than during the normothermia period in the XeNT group 38.2 mmHg (33.1, 40.6) to account for the PCO2 lowering effect of hypothermia “Fig 6”.

**Fig 7:**
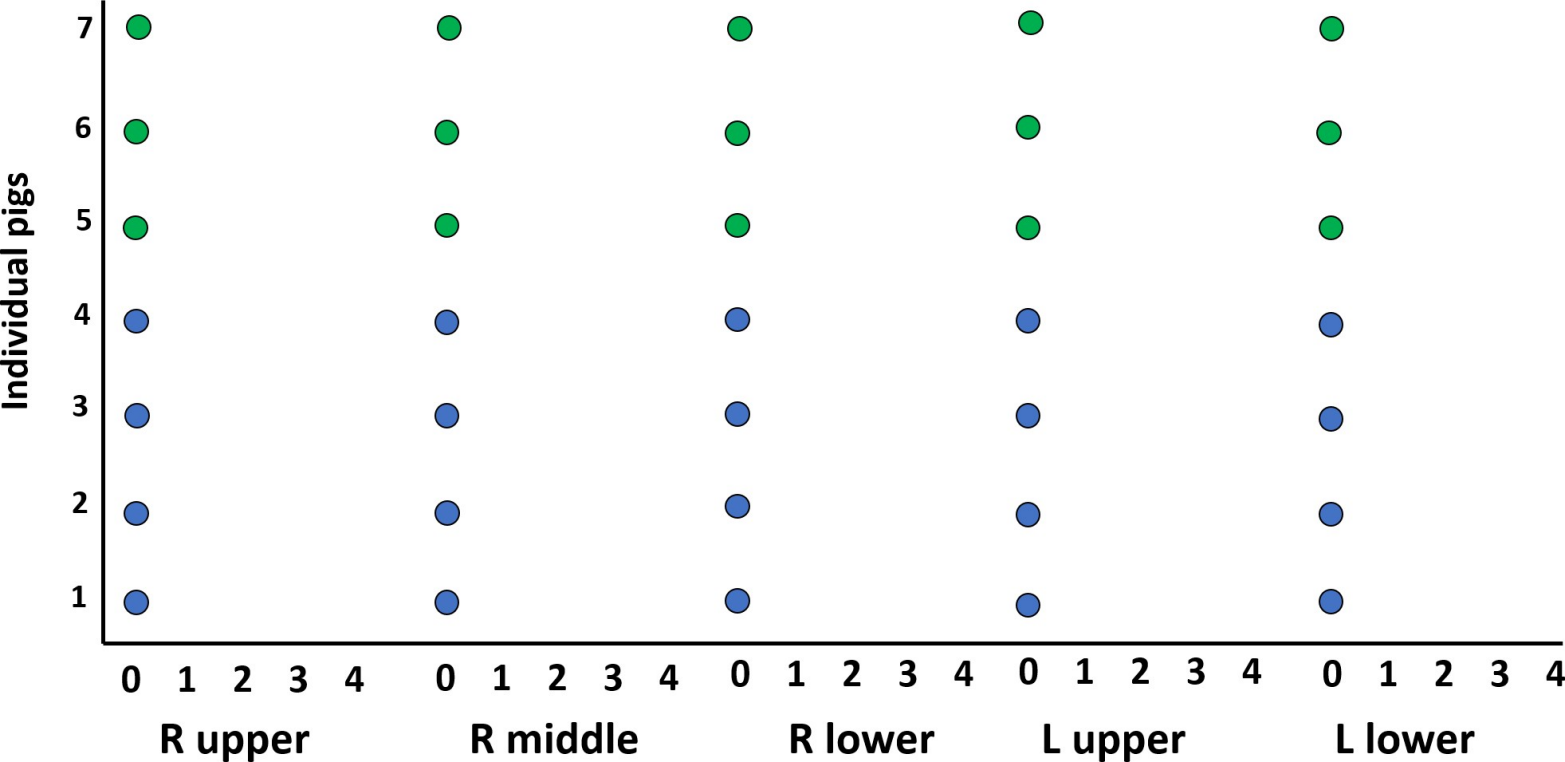
Lung histology scoring from the right and left lung. Lung histopathology scoring includes: no histological abnormalities (0), intraalveolar fibrin (1), hyaline membranes (2), inflammation (3) and oedema (4). Lung lobes are represented as, R upper: Right upper lobe; R middle: Right middle lobe; R lower: Right lower lobe; L upper: Left upper lobe; L lower: Left lower lobe. Blue filled circles represent the XeHT group and the green filled circles represent the XeNT group.

**Fig 8:**
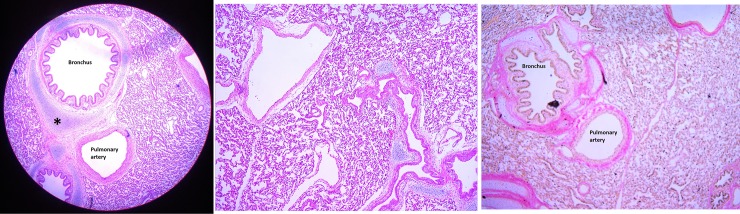
Lung histology indicating no evidence of injury. A] haematoxylin & eosin stained low power microscopic section (* hyaline cartilage plate) B] haematoxylin & eosin stained section showing normal alveolar architecture with no evidence of interstitial oedema, fibrosis, haemorrhage or haemosiderin-laden macrophages and C] Verhoeff’s Van Gieson stained section for elastic fibres. The vasculature showed normal appearances.

With respect to lung pathology, there were no macroscopic injury noted during the autopsy. There were no histologic features indicating lung injury in the right upper, middle and lower lobes and in the left upper and lower lobes “Fig 7”. None of the animals had intra-alveolar fibrin, hyaline membranes, inflammation and oedema. There was no evidence of atelectasis. Eosin and haematoxylin stained sections from three different animals are shown in “Fig 8”.

## Discussion

The majority of measured xenon concentrations fell within 20% variation of the set target value; 8830 out of 9626 total measurements (92%). The majority of measured oxygen concentrations fell within 20% variation of the set target value; 6630 out of 9635 total measurements (69%).

We evaluated the control system performance using previously described methods used to evaluate other closed loop control systems [39,40]. We analysed the performance of the control system with respect to the period of xenon/oxygen delivery in each individual animal and then also the overall performance with respect to the pooled data from all the animals. The pooled data analysis gives a weight to each measurement according to the total number of measurements obtained for each of the seven animals. We did this because averaging the measurements would underemphasise data from piglets with fewer xenon and oxygen measurements while overemphasising the data from the others with a larger number of gas concentration measurements. In each of the seven animals, we delivered xenon for three days to replicate the clinical scenario where xenon might be applied for the entire cooling period currently recommended for clinical use. From the perspective of the gas delivery and control system evaluation, this therefore represented 21 days of use which we regarded as sufficient for an initial technical evaluation.

The negative MDPE, a measure of bias, shows that overall, the xenon concentration was maintained slightly below target levels by a median of 3.3% while the oxygen concentration was maintained slightly above the target values by a median of 9.6%. The MDAPE variable reveals that our system resulted in xenon concentration values that differed from target by a median of 4.3% (of the target value) while with respect to oxygen concentrations the median value was 12.4%. This reflects the accuracy of the system with respect to the set target gas concentration for each gas. The outliers in the absolute performance error and wobble capture the incidents indicated in Table 1 and the 8 hourly manual partial flushes of the system, during which the measured gas concentrations come down significantly followed by achieving the target concentration with time.

None of the pigs showed any evidence of injury to the lung parenchyma or lung vasculature. This is reassuring as despite administration of xenon, a gas with a density 4.5 times that of air, for long periods there was no microscopic evidence of lung injury. The lung sections from each lobe were qualitatively assessed by a pathologist who was blinded to the intervention. Although, a quantitative assessment was not conducted, we are confident that the qualitative technique which is used in clinical practice will pick up any clinically significant injury to the lungs.

Transcutaneous oxygen saturation was recorded every minute. A clinically significant desaturation occurred in 0.1% of these. Some of these desaturations coincided with the endotracheal tube occlusion episodes and other practical problems requiring clinical intervention described in Table 1. Furthermore, the quality of pulse waveform during these low values were unable to be checked offline and movement artefact affecting the values could also not be excluded. This low level of clinically significant desaturation is comparable to those seen in intensive care settings, where appropriate interventions would be instituted as had happened in our experiments and so long as they are recognised and dealt with promptly, they would be unlikely to impact the brain recovery.

There were a number of technical challenges in this investigation. Since the set target value for xenon was typically twice the set target value for oxygen then any given numerical deviation in the measured fraction of each gas in the breathing system from target value will appear to be greater when expressed as a percentage of the target value with respect to oxygen than it would with respect to xenon. It is technically easier to accurately control the concentration of xenon in a binary xenon/oxygen mixture where xenon comprises approximately half the gas in the system, with the balance simply being made up of oxygen. However, in neonates with HIE who are at risk of worsening the brain injury due to even mild hyperoxia, it is considered undesirable to deliver an oxygen fraction higher than absolutely necessary. This becomes challenging if the user set target oxygen fraction approaches 0.21 as with any automated control system, the measured concentration will vary not only slightly above, but also slightly below, the target value over time. Setting the F_i_O_2_ to a default target of 0.33 for example to allow for such variations would not always be clinically acceptable post-hypoxia in neonates. In contrast, with respect to xenon and a typical set target of 0.5, such a variation would be clinically less important. A further challenge for this experiment was that the controller had to simultaneously control the concentration of xenon, representing approximately half of the gas in the system, as well as that of oxygen, representing approximately a quarter of the gas in the system, and nitrogen as the balance gas, complicated by the fact that injection of any one gas under computer control would dilute the concentration of the others present.

It can be seen that the automated control system performed better than these pooled values when data from individual runs are examined. Those with the lowest overall xenon consumption tended to be the piglets where there were fewer technical clinical problems over the 72h period that needed manual intervention, for example breathing system leaks which the controller was then trying to compensate for and tracheal tube partial occlusion with underventilation, among others. This suggests that the overall performance of the controller system might be improved if the incidence of technical and clinical problems not directly due to the controller algorithm itself could be reduced as clinical experience developed.

For example, with respect to two of the incidents summarised as part of Table 1, Pig 3 experienced several periods of ventilation difficulty, even after the tracheal tube had been replaced, requiring multiple episodes of suction and lavage via the tracheal tube. During this period this would have negatively impacted upon the ability of the gas controller to accurately control the breathing gas mixture as a normal tidal volume is desirable to circulate and evenly mix any injected fresh gases before an updated measurement of the gas concentrations is made by the sensors.

The small tidal volume and reduced respiratory rate requirements of piglets weighing 1.53kg i.e. half that of a human neonate, once cooled, would reduce the rapidity of even gas mixing through the circle after each fresh gas injection. Although ameliorated by using the mean of multiple gas concentration measurements separated in time, it might be expected that in larger human neonates this possible source of error would be reduced.

In the best experiment (Piglet 1) the xenon consumption was only 0.15Liters ($ 4.5) per hour and in this case it was notable that there were also the least number of clinical and technical problems. This example perhaps suggests what might be achievable with further technical development and also practical clinical experience.

## Conclusions

In conclusion we have demonstrated that using an automated closed-circuit xenon delivery system, it is feasible to administer xenon consistently at a pre-determined therapeutic concentration for 72 to 78 hours in conjunction with hypothermia at a potentially low cost without inducing lung injury. The challenges faced in the study were largely unrelated to the system design suggesting that with further development an automated control system could have a place where xenon is delivered for long periods in human neonatal research trials.
